# Supplementary figures and images for: Yeast NDI1 reconfigures neuronal metabolism and prevents the unfolded protein response in mitochondrial complex I deficiency
Source: PLoS Genet. 2023 Jul 3;19(7):e1010793. doi: 10.1371/journal.pgen.1010793 (PMC10348588; doi:10.1371/journal.pgen.1010793)

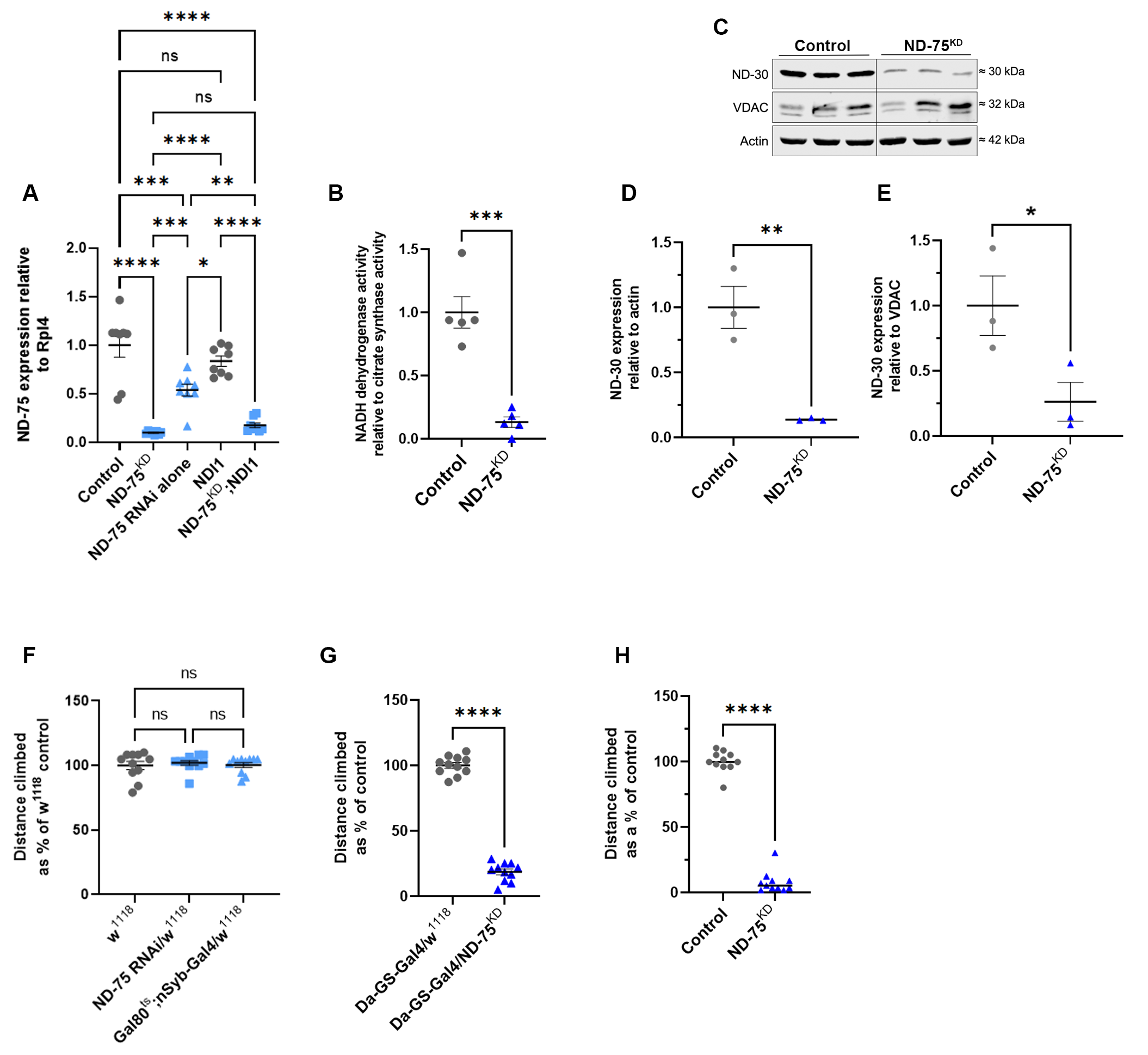

Supplement: S1 Fig — (A) qRT-PCR analysis of ND-75 mRNA levels from adult flies with ubiqitious ND-75KD using Da-GS-Gal4. n = 8 biological replicates for all genotypes. (B) Complex I activity in mitochondria isolated from adult flies with ubiqitious ND-75KD using Da-GS-Gal4. Control n = 5, ND-75KD n = 5 biological replicates. (C) Western blot analysis of ND-30 expression in adult flies with ubiqitious ND-75KD using Da-GS-Gal4. (D, E) Quantification of ND-30 expression relative to actin (D) and the mitochondrial outer membrane protein VDAC (E). Control n = 3, ND-75KD n = 3 biological replicates. (F) Heterozygous ND-75 RNAi flies do not have a climbing phenotype. n = 11 for all genotypes. (H) (G) Ubiqitious ND-75KD using Da-GS-Gal4 causes a strong climbing phenotype. Control n = 11, ND-75KD n = 11. (H) Reduced climbing abilty of flies expressing an alternative ND-75 shRNA (HMS00854) in neurons with Tub-Gal80ts; nSyb-Gal4. Control n = 11, ND-75KD n = 11. Controls are Da-GS-Gal4 or Tub-Gal80ts; nSyb-Gal4 hemizygotes. 5 day old male and female flies were used in (A)-(E). 1 day old male flies were used in (F, H). 5 day old male flies were used in (G). Data are represented as mean ± SEM and were analysed using Student’s unpaired t-test. ns not significant, *p<0.05, ***p < 0.001, ****p < 0.0001. (TIF) [file pgen.1010793.s001.tif]

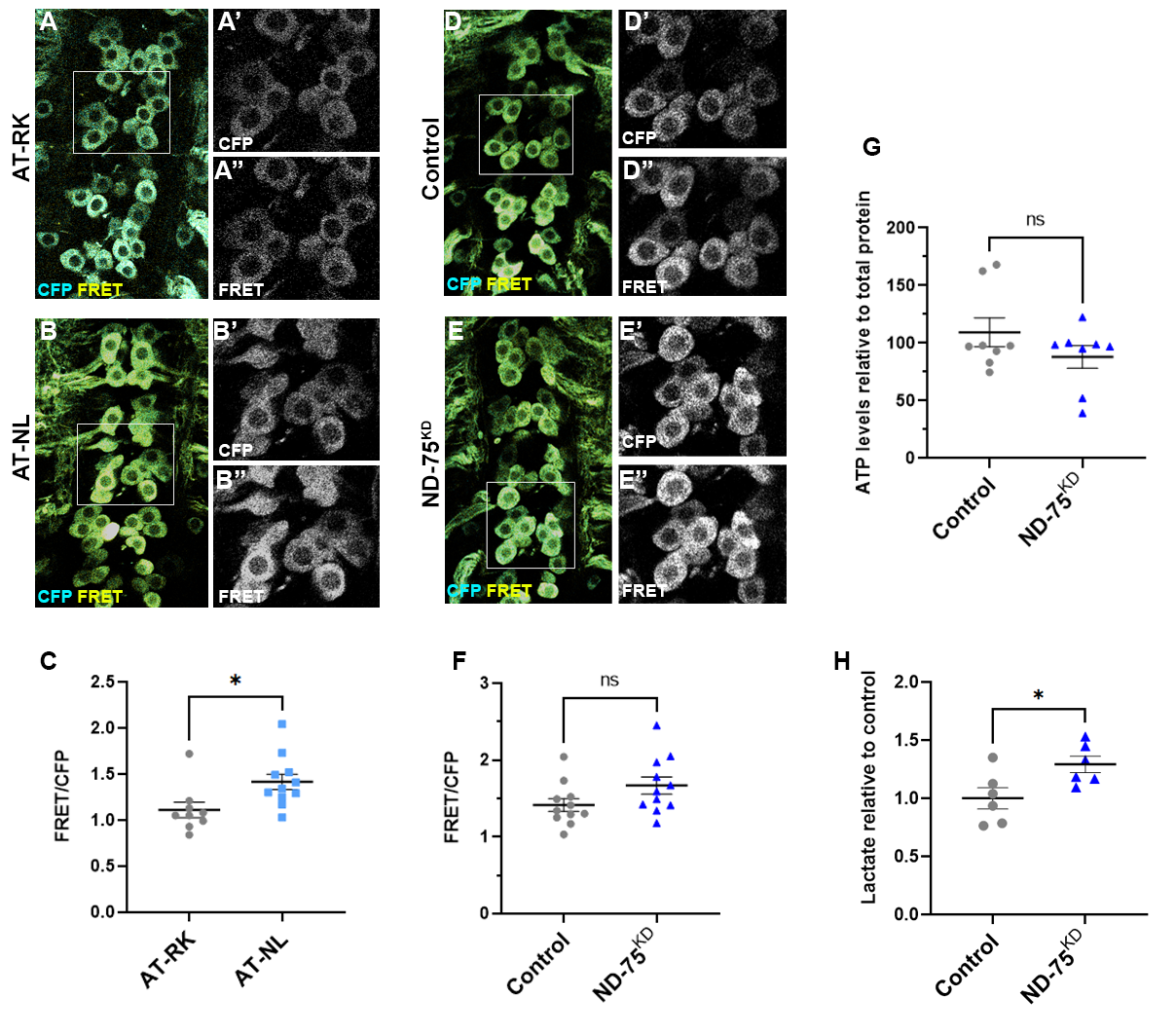

Supplement: S2 Fig — (A-B”) Fluorescence emission of a control ATP insensitive probe (AT-RK) (A-A”) and the ATP-sensing AT-NL probe (B-B”) when excited at 405nm (CFP, cyan) and 488nm (FRET, yellow) in larval motor neurons using OK371-Gal4. (C) Quantification of CFP/FRET fluorescence. Control n = 5, ND-75KD n = 5 larval CNS. (D-E”) AT-NL fluorescence emission of control and ND-75KD larval motor neurons when excited at 405nm (CFP, cyan) and 488nm (FRET, yellow) using OK371-Gal4. AT-RK n = 9, AT-NL n = 11 larval CNS. (F) Quantification of CFP/FRET fluorescence in control and ND-75KD neurons. Control n = 11, ND-75KD n = 11 larval CNS. (G) ATP levels measured in whole heads from control flies or with with pan-neuronal ND-75KD using Tub-Gal80ts; nSyb-Gal4. Control n = 8, ND-75KD n = 8 biological replicates. (H)Lactate levels from metabolomic analysis of pan-neuronal ND-75KD using Tub-Gal80ts; nSyb-Gal4. Control n = 6, ND-75KD n = 6.. 2 day old male and female flies were used in (G) and (H). Controls are OK371-Gal4 or Tub-Gal80ts; nSyb-Gal4 hemizygotes. Student’s t test. Data are represented as mean ± SEM. ns not significant, *p < 0.05. (TIF) [file pgen.1010793.s002.tif]

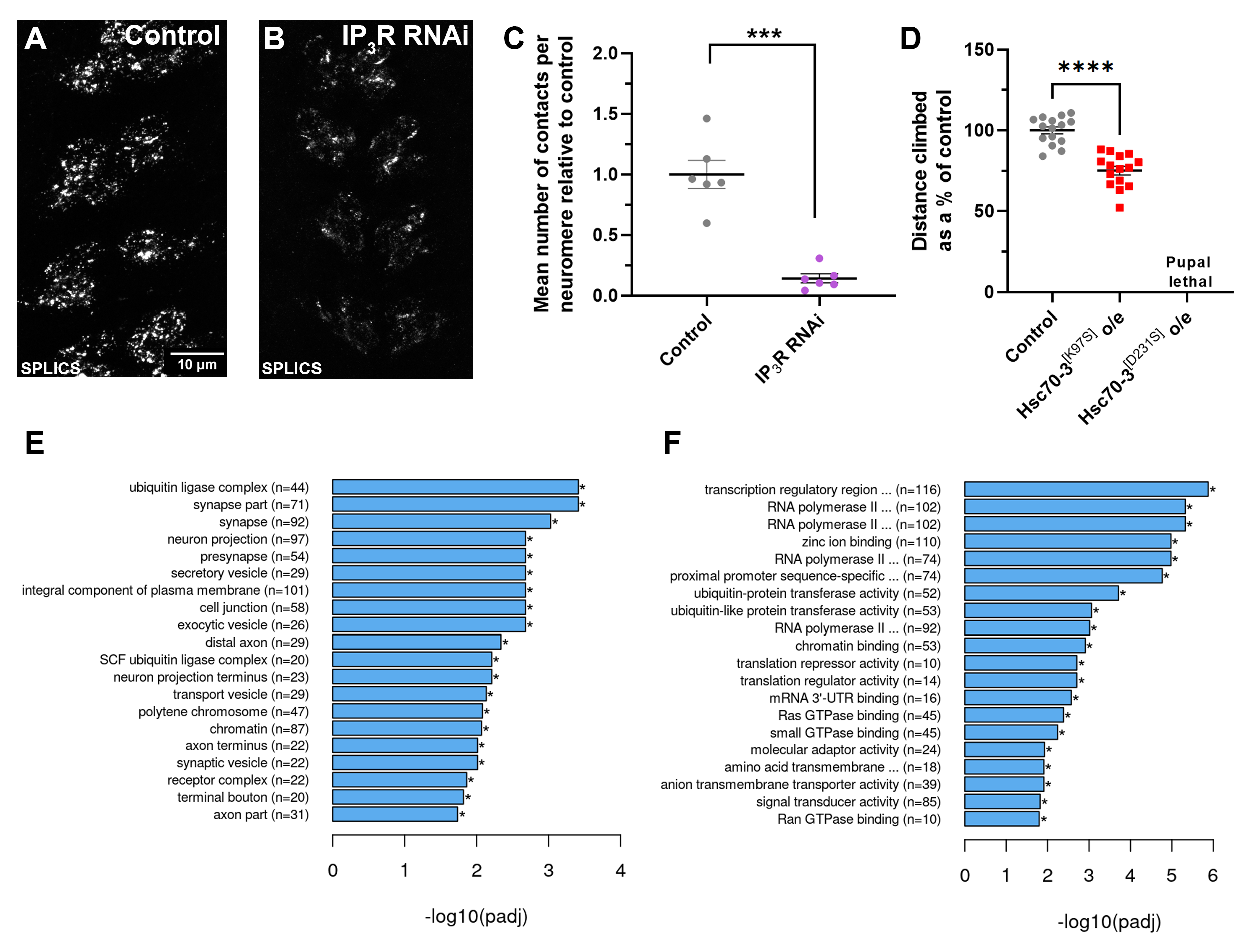

Supplement: S3 Fig — (A, B) Visualisation of ER-mitochondria contacts by SPLICS expression in larval motor neurons using OK371-Gal4 in control (A) and with IP3R knockdown (B). (C) Quantification of ER-mitochondria contacts. Control n = 6, IP3R n = 6 larval CNS. (D) Climbing ability of flies with Hsc70-3[K97S] and Hsc70-3[D231S] overexpression in neurons using nSyb-Gal4. Control n = 15, Hsc70-3K97S n = 15. Controls are OK371-Gal4 or nSyb-Gal4 hemizygotes. Data are represented as mean ± SEM and were analysed using Student’s t test. ***p < 0.001, ****p < 0.0001 (E, F) GO cellular component (E) and molecular function (F) analyses of genes with significantly increased expression in head tissue from pan-neuronal ND-75KD using Tub-Gal80ts; nSyb-Gal4. (TIF) [file pgen.1010793.s003.tif]

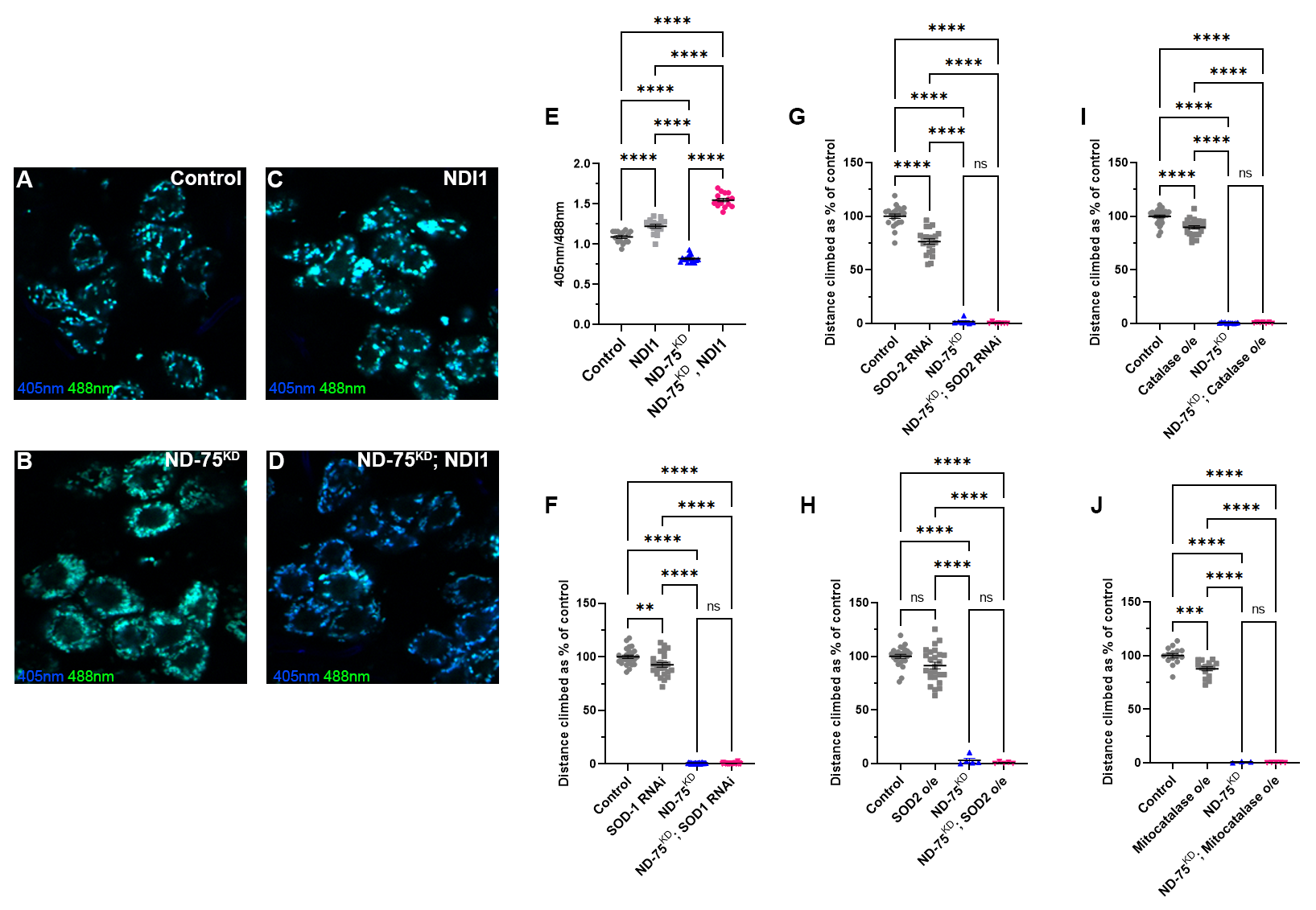

Supplement: S4 Fig — (A-D) mito-roGFP2-Grx1 expression showing merge of 405nm excitation (blue) and 488nm excitation (green) emission fluorescence in control (A) and ND-75KD (B), NDI1 expression (C) and or ND-75KD with NDI1(D) larval motor neurons using OK371-Gal4. (E) Quantification of mito-roGFP2-Grx1 405nm and 488nm excitation fluorescence. n = 15 larval CNS for all genotypes. (F-J) Climbing ability of pan-neuronal ND-75KD, using Tub-Gal80ts; nSyb-Gal4, with: (F) SOD1 RNAi, control n = 25, SOD1 RNAi n = 24, ND-75KD n = 24, ND-75KD;SOD1 RNAi n = 22 flies. (G) SOD2 RNAi, control n = 21, SOD2 RNAi n = 21, ND-75KD n = 7, ND-75KD;SOD2 RNAi n = 7 flies. (H) SOD2 overexpression (o/e), control n = 25, SOD2 o/e n = 25, ND-75KD n = 5, ND-75KD;SOD2 o/e n = 5 flies. (I) catalase overexpression (o/e), control n = 27, catalase o/e n = 24, ND-75KD n = 11 ND-75KD; catalase o/e n = 7 flies. (J) mitocatalase overexpression (o/e), control n = 15, mitocatalase o/e n = 15, ND-75KD n = 3 ND-75KD; mitocatalase o/e n = 5 flies. Controls are OK371-Gal4 or Tub-Gal80ts; nSyb-Gal4 hemizygotes. 1 day old male flies were used in (G-J). Student’s t test. Data are represented as mean ± SEM. n.s. not significant, *p < 0.05, **p<0.01, ***p<0.001, ****p < 0.0001. (TIF) [file pgen.1010793.s004.tif]
