# Supplementary material for: Yeast NDI1 reconfigures neuronal metabolism and prevents the unfolded protein response in mitochondrial complex I deficiency
Source: PLoS Genet. 2023 Jul 3;19(7):e1010793. doi: 10.1371/journal.pgen.1010793 (PMC10348588; doi:10.1371/journal.pgen.1010793)
Supplement: S8 Table — (DOCX) [file pgen.1010793.s012.docx]

| **Stock** | **Full genotype** | **Source & stock number** | **Construct ID** | **Reference** |
| --- | --- | --- | --- | --- |
| *w^1118^* | *w[1118];+/+;+/+;+/+* | BDSC 6326 |  |  |
| *nSyb-Gal4* | *y[1] w[*]; +/+; P{nSyb-GAL4.S}3; +/+* | BDSC 51635 |  |  |
| *UAS-Dcr2;OK371-Gal4,UAS-CD8-GFP* | *UAS-Dcr2;OK371-Gal4,UAS-CD8-GFP; +/+;+/+* | From Darren Williams, King’s College London |  | [1] |
| *UAS-PERK RNAi* | *y[1] v[1]; P{y[+t7.7] v[+t1.8]=TRiP.HMJ02063}attP40; +/+;+/+* | BDSC 42499 | HMJ02063 | [2] |
| *Daughterless GeneSwitch-GAL4* | *w[*]; P{da-GSGAL4.T};+/+;+/+* | Nazif Alic, University College London |  | [3] |
| *Tubulin-GAL80^ts^* | *w[*]; P{w[+mC]=tubP-GAL80[ts]}10; TM2/TM6B, Tb[1];+/+* | BDSC 7108 |  |  |
| *UAS-ND-75 RNAi* | *y[1] sc[*] v[1] sev[21]; +/+;P{y[+t7.7] v[+t1.8]=TRiP.HMS00853}attP2;+/+* | BDSC 33910 | HMS00853 | [2] |
| *UAS-ND-75 RNAi* | y[1] sc[*] v[1] sev[21]; +/+;P{y[+t7.7] v[+t1.8]=TRiP.HMS00854}attP2;+/+ | BDSC 33911 | HMS00854 | [2] |
| *UAS-mitoGFP* | *w[1118]; P{w[+mC]=UAS-mito-HA-GFP.AP}2/CyO;+/+;+/+* | BDSC 8442 |  | [4] |
| *UAS-SPLICS_L_* | *w[*];+/+;UAS-SPLICS_L_;+/+* | This study |  |  |
| *UAS-IP_3_R RNAi* | *y[1] v[1]; P{y[+t7.7] v[+t1.8]=TRiP.GLC01786}attP40;+/+;+/+* | BDSC 51686 | GLC01786 | [2] |
| *UAS-Hsc70-3^K97S^* | *w[126]; P{w[+mC]=UAS-Hsc70-3.K97S}D;+/+;+/+* | BDSC 5842 |  |  |
| *UAS-Hsc70-3^D231S^* | *w[126]; P{w[+mC]=UAS-Hsc70-3.D231S}D;+/+;+/+* | BDSC 5841 |  |  |
| *UAS-NDII* | *w[*];+/+;UAS-NDI1;+/+* | Alex Whitworth, MRC Mitochondrial Biology Unit, Cambridge |  | [5] |
| *UAS-ATeam1.03NL (AT-NL)* | *w[*]; P{w[+mC]=UAS-AT1.03NL}1;+/+;+/+* | Kyoto DGGR 117011 |  | [6] |
| *UAS-ATeam1.03RK (AT-RK)* | *w[*]; P{w[+mC]=UAS-AT1.03RK}1 / CyO;+/+;+/+* | Kyoto DGGR 117013 |  | [6] |
| *UAS-Sod-1 RNAi* | *w[1118]; P{KK102426}VIE-260B;+/+;+/+* | VDRC 108307 | KK102426 |  |
| *UAS-Sod-2 RNAi* | *w[1118]; P{KK108954}VIE-260B;+/+;+/+* | VDRC 110547 | *KK108954* |  |
| *UAS-Sod-2* | *w[1118]; UAS-Sod2;+/+;+/+* | From Matthias Landgraf, University of Cambridge. |  | [7] |
| *UAS-Catalase* | *w[1118]; UAS-Catalase;+/+;+/+* | From Matthias Landgraf, University of Cambridge. |  | [8] |
| *UAS-MitoCatalase* | *w[1118]; UAS-MitoCatalase;+/+;+/+* | From Matthias Landgraf, University of Cambridge. |  | [9] |

**References**

1. Cagin U, Duncan OF, Gatt AP, Dionne MS, Sweeney ST, Bateman JM. Mitochondrial retrograde signaling regulates neuronal function. Proc Natl Acad Sci U S A. 2015;112(44):E6000-9. Epub 2015/10/23. doi: 10.1073/pnas.1505036112. PubMed PMID: 26489648.

2. Perkins LA, Holderbaum L, Tao R, Hu Y, Sopko R, McCall K, et al. The Transgenic RNAi Project at Harvard Medical School: Resources and Validation. Genetics. 2015;201(3):843-52. Epub 20150828. doi: 10.1534/genetics.115.180208. PubMed PMID: 26320097; PubMed Central PMCID: PMCPMC4649654.

3. Tricoire H, Battisti V, Trannoy S, Lasbleiz C, Pret AM, Monnier V. The steroid hormone receptor EcR finely modulates Drosophila lifespan during adulthood in a sex-specific manner. Mechanisms of ageing and development. 2009;130(8):547-52. Epub 20090530. doi: 10.1016/j.mad.2009.05.004. PubMed PMID: 19486910.

4. Horiuchi D, Barkus RV, Pilling AD, Gassman A, Saxton WM. APLIP1, a kinesin binding JIP-1/JNK scaffold protein, influences the axonal transport of both vesicles and mitochondria in Drosophila. Curr Biol. 2005;15(23):2137-41. doi: 10.1016/j.cub.2005.10.047. PubMed PMID: 16332540; PubMed Central PMCID: PMCPMC1532932.

5. Sanz A, Soikkeli M, Portero-Otin M, Wilson A, Kemppainen E, McIlroy G, et al. Expression of the yeast NADH dehydrogenase Ndi1 in Drosophila confers increased lifespan independently of dietary restriction. Proc Natl Acad Sci U S A. 2010;107(20):9105-10. Epub 2010/05/04. doi: 0911539107 [pii]

10.1073/pnas.0911539107. PubMed PMID: 20435911; PubMed Central PMCID: PMC2889079.

6. Tsuyama T, Tsubouchi A, Usui T. Mitochondrial dysfunction induces dendritic loss via eIF2alpha phosphorylation. Journal of Cell Biology. 2017;216(3):815-34. doi: 10.1083/jcb.201604065. PubMed PMID: 28209644.

7. Missirlis F, Rahlfs S, Dimopoulos N, Bauer H, Becker K, Hilliker A, et al. A putative glutathione peroxidase of Drosophila encodes a thioredoxin peroxidase that provides resistance against oxidative stress but fails to complement a lack of catalase activity. Biological chemistry. 2003;384(3):463-72. Epub 2003/04/29. doi: 10.1515/bc.2003.052. PubMed PMID: 12715897.

8. Missirlis F, Phillips JP, Jäckle H. Cooperative action of antioxidant defense systems in Drosophila. Curr Biol. 2001;11(16):1272-7. Epub 2001/08/30. doi: 10.1016/s0960-9822(01)00393-1. PubMed PMID: 11525742.

9. Bayne AC, Mockett RJ, Orr WC, Sohal RS. Enhanced catabolism of mitochondrial superoxide/hydrogen peroxide and aging in transgenic Drosophila. The Biochemical journal. 2005;391(Pt 2):277-84. doi: 10.1042/bj20041872. PubMed PMID: 15954861; PubMed Central PMCID: PMCPMC1276925.
