# Supplementary material for: Yeast NDI1 reconfigures neuronal metabolism and prevents the unfolded protein response in mitochondrial complex I deficiency
Source: PLoS Genet. 2023 Jul 3;19(7):e1010793. doi: 10.1371/journal.pgen.1010793 (PMC10348588; doi:10.1371/journal.pgen.1010793)
Supplement: Attachment — Submitted filename: Granat reviews new 2.pdf [file pgen.1010793.s013.pdf]

We are very grateful to the reviewers for their helpful and constructive comments. We have addressed the reviewers' comments by performing additional behavioural analysis, qRT-PCR and complex I assays, as well as additional data analyses, clarification in the text and figures. A detailed list of our responses to the reviewers' comments and description of the changes made to the manuscript are given below in blue. We have also highlighted the modifications to the text of the main manuscript and Supplemental material using track changes.

Reviewer #1: Mitochondrial complex I deficiency causes severe neurological diseases including Leigh syndrome. The pathogenic mechanism of the disease is poorly understood. In this manuscript, the authors modelled complex I deficiency in Drosophila by knocking down the mitochondrial complex I subunit ND-75 (NDUFS1) specifically in neurons. They found that complex I deficiency affects neuronal function and lifespan, without reducing the overall cellular ATP levels. In the mutant flies, they observed increased mitochondrial density, reduced endoplasmic reticulum-mitochondria contacts and activation of the endoplasmic reticulum unfolded protein response (UPR) in neurons. The data also suggested changes to mitochondrial metabolism. Interestingly, expression of the yeast NADH dehydrogenase NDI1, which does not pump protons, corrected some of these metabolic changes, reinstated endoplasmic reticulum-mitochondria contacts, prevented UPR activation and rescued the behavioral and lifespan phenotypes caused by complex I deficiency. Based on these data, the authors concluded that aberrant NAD+/NADH ratio and metabolic disruption in the complex I-deficient neurons drive UPR activation and neuronal pathology.

This is a well executed study. Most of the data (behavioral and biochemical) presented are strong. Particularly, the behavioral and survival defects and altered stress signaling caused by complex I deficiency can be robustly corrected by the yeast NDI1 gene. This suggests that metabolic defect other than energy deficiency may contribute to the loss of neuronal function. Although the exact mechanism remains unidentified, this finding may open a new direction for the better understanding of pathologies caused by complex I-deficiency. The following recommendations may improve the manuscript.

Major recommendations:

1) Given that mitochondria and ER are under stress in complex I-deficient neurons, the data from the split-GFP experiment for evaluating the mito-ER contact sites may be at risk of overinterpretation. Under these conditions, there is no guaranty that the split GFP constructs are efficiently targeted to the organelles. This may confound the interpretation of the data. This part of the data is not critical for the central theme of the manuscript. The authors should consider removing it from the manuscript.

For the following reasons we respectfully disagree with the reviewer and have kept the SPLICS data in the manuscript:

(1) Cieri et al., 2018 (PMID 29229997) showed that the same SPLICS probe detects changes in mito-ER contacts in mammalian cells with severe alterations in mitochondrial morphology and function caused by overexpression of Drp1, expression of a dominant negative Drp1, knockdown of Mfn2 and loss of Parkin. These published data show that the SPLICS probe efficiently detects mito-ER contacts in cells with dysfunctional mitochondria.

(2) In our experiments, SPLICS will be expressed and localised very likely before the ND-75 RNAi has reduced the level of ND-75 protein and before complex I deficiency in neurons.

(3) Although we do not have direct evidence that the split GFP constructs are efficiently targeted to ER and mitochondria in ND-75 KD neurons, our SPLICS data are consistent with the independent quantification of mito-ER contacts using TEM (the 'Gold standard' for quantification of mito-ER contacts) in the larval CNS and adult brain (Figure 2H-J and Figure 6G-K).

2) ATF4 activation and phosphorylation of eIF2alpha are increased in ND-75KD flies. The authors assume that the (classic ER) UPR is activated. How can this be distinguished from ISR activation that is known to be stimulated by mitochondrial dysfunction? In this context, although the dominant negative alleles of Hsc70-3 cause pupal lethality and climbing defects, there is no guaranty that this entails the same physiological consequences as complex I deficiency. As such, the conclusion that "This finding strongly suggests that activation of the UPR contributes to neuronal dysfunction in ND-75KD flies" is weak. Accordingly, the authors may consider reword the title.

The reviewer may have missed this, as it is described briefly in the Results on p.8, but we show in Figure 3D and quantify in Figure 3J that ND-75 KD induced activation of ATF4 is completely abrogated by simultaneous knockdown of PERK. These data clearly demonstrate that ATF4 activation in ND-75 KD neurons is due to activation of the UPR. We appreciate the criticism of our interpretation of the Hsc70-3 data and have modified the wording accordingly in the revised manuscript on p.8.

3) It looks that mitochondrial density is increased in ND-75KD flies. There are no data supporting morphological changes to mitochondria. The authors need to reword the text. On the other hand, increased mitochondrial density is not consistent with the overall repression of nuclear genes encoding mitochondrial proteins. Any explanation for this discrepancy?

We have now included analysis of mitochondrial volume shown in Figure 2D (and Figure 6F) and described on p.6 in the revised manuscript. We don't know why genes encoding mitochondrial proteins are repressed when mitochondrial number and volume are increased but this is something we plan to investigate in future.

4) To exclude the possibility that the ectopic expression of NDI1 induces compensatory adaptation to mitochondrial damage independent of NADH dehydrogenase activity, it would be important to show the levels of NADH dehydrogenase in the rescued flies. Either positive or negative data would be helpful for better understanding how neuronal function is affected.

We have now analysed complex I activity in flies expressing NDI1 alone and NDI1 together with ND-75 KD and find that NDI1 expression rescues the loss of NADH dehydrogenase activity caused by ND-75 KD. These data are now shown in Figure 5A and described on p.11 of the revised manuscript.

Minor points:
1) The "drastic" changes in the expression of genes encoding the ETC and TCA cycle are embedded in the supplemental tables that are not easy to navigate by the readers. It would be good if the authors can make a figure illustrating the top hits and show it in one of the figures.

This is an excellent suggestion and so in Figure 4 we have now included a heat map showing the decreased expression of ETC and TCA cycle genes in ND-75 KD flies (Figure 4C in the revised manuscript).

2) Page 4, paragraph 2 – "Importantly, targeting stress signaling pathways, including the UPR and ISR, has shown therapeutic potential in animal models". It would be good if the authors can cite the relevant references.

We have now cited relevant references here in the revised manuscript on p.4.

3) The asterisks denoting the statistical values in the figures throughout the manuscript need to be annotated differently as they are all too small and sometime difficulty to differentiate from the data points.

We have now added lines or brackets between pairwise comparisons and increased the size of the asterisks in all the figures.

4) Page 6, paragraph 3 – delete "have".

We deleted this word on p.6.

5) Page 36; Figure S2 – (H) panel is missing.

Thanks for spotting this, we have corrected it in the legend for Supplemental Figure S2 in the revised manuscript.

Reviewer #2: CI deficiency leads to severe neuropathologies and childhood mortality. In this study, Granat et al model CI deficiency in Drosophila by neuron-specific RNAi of the 75kDa subunit (NDUFS1). The authors successfully exploit the benefits of Drosophila as an in vivo system, and show convincingly that the KD flies exhibit severe neurological dysfunction (climbing, locomotion, seizures), which impacts on their survival, and provide evidence implicating the UPR in this process. Overall, this is an interesting and well written manuscript, which will have broad appeal and relevance to many fields (mitochondria, neurodegeneration, metabolism, fly models of genetic disease).

While the data presented are clear and compelling, there are several points that would benefit from being addressed or discussed prior to publication.

1) Control lines - Throughout the manuscript, 'control' refers to the driver line alone. For selected experiments, it would be helpful to have the additional UAS-RNAi alone control (e.g. q-RT-PCR in Fig S1A). This would confirm that the transgene is not leaky i.e. expressed in the absence of the driver, which would be important for conclusions relating to the tissue-selectivity of subsequent manipulations.

We have now performed qRT-PCR of the hemizygous ND-75 UAS-RNAi alone control flies and find a significant (~50%) reduction in ND-75 expression compared to the driver alone control (Supplemental Figure S1A), indicating the transgene is somewhat 'leaky'. However, the climbing ability of ND-75 UAS-RNAi alone control flies is the same as hemizygous driver alone and w[1118] controls. By contrast ubiquitous ND-75 KD in adults causes a strong climbing phenotype. Therefore, any leakiness is very unlikely to contribute to the severe ND-75 KD phenotypes. These data are described in the Results on p.5 and shown in Supplemental Figure S1A, F, G in the revised

manuscript.

Similarly, there may be effects due to differences in genetic backgrounds between experimental groups. The TRIP collection includes several lines that could be used as more appropriate background controls (https://fgr.hms.harvard.edu/trip-rnai-control-fly-stocks).

We appreciate this suggestion and we have confirmed that Gal4 driver crossed to w[1118] control climbing and lifespan phenotypes are similar to Gal4 driver crossed to UAS-lacZ TRIP RNAi (see below). Therefore, we did not feel it was worth repeating all the experiments in the manuscript using a control RNAi line. However, we are using UAS-lacZ and UAS-mCherry TRIP lines as controls in current and future experiments.

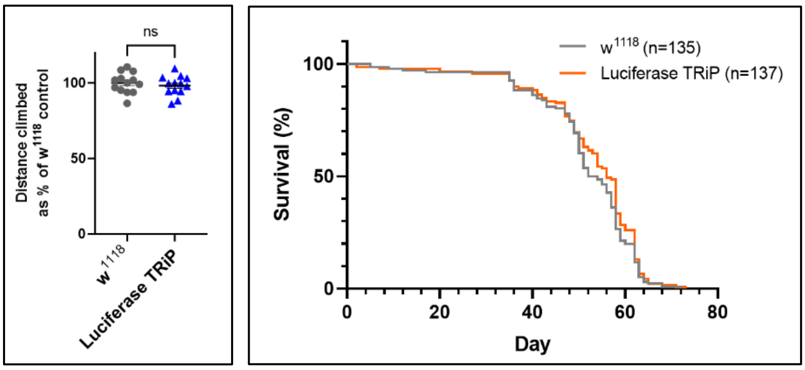

Relating to Figs 4-6, it would be valuable to confirm if the level of ND-75 knock-down is the same when the UAS-RNAi line is combined with the additional UAS-Ndi1 line.

We have now performed qRT-PCR analysis of ND-75 expression in flies with the UAS-ND-75 RNAi and the additional UAS-Ndi1 line. These data show that UAS-Ndi1 does not alter the strong reduction in ND-75 expression caused by ND-75 RNAi. These data are described in the Results on p.10 and shown in Supplemental Figure S1A of the revised manuscript.

2) Mitochondrial morphology - Morphological changes to mitochondria are evident from the mitoGFP imaging, but based on Figs 2A-B and given their network-like nature, it is not entirely clear how a ‘number of mitochondria’ was reached. Would area/volume be more appropriate?

We have now included analysis of mitochondrial volume shown in Figures 2D and 6F and described on p.6 and p.11 in the revised manuscript. Mitochondrial number and volume were quantified using the Measurement tool in Volocity (see Materials and Methods).

3) Experimental samples (sex, age, temperature) - Regarding the samples used for analysis, many of the experiments appear to pool male and female flies. The rationale for this mixing is unclear, since many metabolic, physiological and behavioural traits (e.g. feeding, activity, survival) have been widely shown to be sex-dependent in flies. Therefore, pooling may introduce biological variability or mask potentially interesting sex-specific effects? For the climbing assays, it is not clear if this was done on male and/or female flies? For the lifespans, which were done only in males, do the authors observe a similar decrease in survival of female flies?

Wherever possible we used males and females to model complex I deficiency as patients of both sexes are similarly affected by the disease. For Drosophila behavioural paradigms that are well

established to be sexually dimorphic (climbing, open field and lifespan) we used males to reduce the variability caused by combining males and females. For seizure and feeding assays we have not observed differences between males and females in control or ND-75 KD flies and so combined males and females. Similarly, for qRT-PCR, CI, metabolomic and transcriptomic analyses we combined males and females. We have now stated that male flies were used for the climbing assays in the Materials and Methods on p.14 of the revised manuscript. For lifespan, although we have not quantified this, we have observed that female ND-75 KD flies have a similarly severely reduced lifespan to male ND-75 KD flies. We have also now stated the sex of the flies used for each experiment in all the figure legends.

Given the severe shortening of lifespan in the ND-75 KD flies (median of 5 days for males), it is understandable that experiments need to be performed on very young flies. However, the age of the flies used is not entirely clear. For example, the RNA-seq is described as being performed on 2 day old flies. Whereas for some experiments, flies are described in the methods as being used the day after eclosion. Are these the same age i.e. do the authors consider the day of eclosion as day 0 or day 1? Since flies are still in the process of maturing during this early period (e.g. the gut, which may potentially impact some of their metabolic readouts), it would be important to know the age of samples. This would help in terms of comparison with other studies and future data reproducibility.

Thanks for pointing this out. We consider the day of eclosion as day 0. Climbing, seizure and feeding assays were performed on the day after eclosion (day 1), while RNA-seq, metabolomics and TEM were performed using 2 day old flies. We have clarified this in the Materials and Methods of the revised manuscript. RNA-seq, metabolomics and TEM were performed using fly heads or brain tissue so would not be affected by non-head e.g. gut tissue.

For the Geneswitch experiments, it was not clear why the flies were incubated for 5 days at 29ºC (since this is not a temperature-sensitive driver)? It would be helpful to explain the rationale, as all other adult experiments appear to have been performed at 25ºC.

Although Geneswitch Gal4 is not acutely temperature sensitive in the same way as, for example, Gal80[ts], the Gal4 protein is more active at 29°C (closer to 30°C, the optimum growth temperature for budding yeast) than 25°C. We therefore performed the Geneswitch experiments at 29°C to maximise Gal4 activity. We have now mentioned this in the Materials and Methods on p.14 of the revised manuscript.

Overall, the exact type of sample (sex & age) should be fully defined in the figure and/or legend, not just in the methods section, which would help facilitate data interpretation.

In addition to the Materials and Methods, we have now fully defined the sex and age of the flies used in all the figure legends in the revised manuscript.

4) Fly strains - Regarding the fly strains used in the study, in addition to the simplified stock name, please state the full genotype including genetic background for each line (Table S5). Also, it would be helpful to include original publication references for any lines that are not available in stock centres. Regarding the ND-75 RNAi lines, please mention more explicitly in the manuscript that these are from the TRIP collection (PMID 26320097).

We have now included the full genotypes including the genetic background and the original publication for lines not available in stock centres in Supplemental Table S8 in the revised manuscript. We have also stated that the ND-75 RNAi lines were from the TRiP collection in the Materials and Methods on p.13 of the revised manuscript.

5) Protein quantification - The VDAC band intensity in Fig S1C appears to be highly variable between samples, which complicates the relative quantification in Fig S1E. Could the authors speculate on this? Regarding CI quantification, the data indicate loss of the whole complex. Have the authors considered potential changes to other complexes in the mitochondrial ETC?

We also noticed the variability in VDAC levels between samples. This may be because VDAC is an outer membrane protein and may suffer from variation in mitochondrial membrane solubilisation during sample preparation.

We have wondered whether there are changes in other complexes in the mitochondrial ETC in ND-75 KD flies, which would be suggested from the RNA-seq data. This is something we plan to investigate in detail in future grant applications based on this study.

6) A previous study has shown that ubiquitous RNAi of CI subunits can extend fly lifespan (PMID 19747824). Could the authors comment on how their neuronal-specific data relate to these findings? Did the authors try lifespans for their ND-75 KD with the inducible ubiquitous daGS driver?

Consistent with our study, Copeland et al 2009 (PMID 19747824) found that ubiquitous knockdown during development of the majority of CI subunits they tested, including ND-75, caused lethality or shortened lifespan. However, as the reviewer points out, knockdown of some CI subunits either during development or in adults caused lifespan extension. For the CI gene CG9172 (NDUFS7) they observed "a threshold effect where moderate knockdown resulted in life extension and stronger inhibition was detrimental, causing developmental lethality". ND-75 KD flies have very strong knockdown in ND-75 expression levels (Supplemental Figure S1), consistent with the detrimental effects of strong CI subunit knockdown observed by Copeland et al.

We have not analysed the lifespan of ND-75 KD with the inducible ubiquitous daGS driver, as we focus on phenotypes caused by neuronal specific knockdown in this manuscript, but we do find a strong reduction in climbing ability in these flies (now shown in Supplemental Figure S1G and described on p.5), which shows that adult specific ubiquitous knockdown of ND-75 is detrimental.

Reviewer #3: The manuscript by Granat et al. describes the effects of complex I subunit ND-75 (NDUFS1) knockdown in Drosophila neurons. ND-75 is a core and the largest subunit of complex I, and its loss resulted in severe motor dysfunction, neurological phenotypes, and reduced lifespan. Molecularly ND-75 knockdown in neurons caused changes in cellular metabolism, transcriptional response, ER-mitochondria contact sites, mitochondrial morphology and UPR. The authors aimed to rescue these phenotypical and molecular phenotypes by expressing the yeast NADH dehydrogenase NDI1 in neurons, which led to almost a complete reversal of the observed parameters.

Overall, the findings are interesting and highlight the importance of NADH dehydrogenase activity of complex I in neurological mitochondrial dysfunction. It is a nice addition to the growing number of studies linking mitochondrial dysfunction to the ISR in a mitochondrial defect- and metabolic state-specific manner.

The authors utilized a variety of imaging techniques and multi-omics approaches. Moreover, the manuscript is clearly written and easy to follow. There are, however, some loose ends that needs tightening. A number of comments and suggestions to improve the study include:

1) ND-75KD flies show an extreme lifespan reduction, with a median survival of 5 days. Hegde et al.'s global and neuronal knockdowns of ND-75 in flies, on the other hand, resulted in median lifespans of

27 and 25 days, respectively. These differences in survival and these models in general should be discussed.

Hegde et al., used a different RNAi line (from the VDRC) to the line we used (from the TRiP collection), which causes much weaker phenotypes. We have now discussed the differences in survival in the two models in the Discussion of the revised manuscript on p.12.

2) Some mitochondrial dysfunction fly models have been shown to have severe feeding difficulties (i.e., dNDUFS4-knockdown flies). Considering the severe neurological phenotypes of the ND-75KD flies, have the authors checked food intake or observed any feeding difficulties in the animals? Starvation could also increase various stress pathways.

This is a very good suggestion. We have now performed a feeding assay and find that ND-75 KD flies have significantly reduced food consumption. These data are described in the Results on p.6 and shown in Figure 1H in the revised manuscript.

3) The results presented in Figure S2 and related to ATP levels are somewhat surprising considering the complex I decrease. For example, dND2 mutant flies, like the current ND-75KD flies, display many of the hallmarks of mitochondrial diseases, including reduced lifespan and signs of neurodegeneration. However, dND2 flies have lower levels of ATP.

The ATP levels in the adult heads of ND-75KD flies show a tendency towards a decrease (Figure S2G). The sample size is not indicated in the figure legends, but this experiment might benefit from increasing the sample size. One would argue that increased glycolysis might also account for the unchanged ATP levels. In that case, this claim should be supported by experimental evidence.

dND2 mutant flies have been shown to have lower ATP levels in heads from 35 day old flies, whereas ATP levels in young dND2 flies were not analysed (PMID: 25085991). ATP levels in ND-75 KD flies were analysed in very young (1 day old) flies, at which time the brain may have more metabolic plasticity than in old flies. Pairwise comparison of the metabolomic data shows a significant increase in lactate levels in ND-75 KD fly heads, suggesting that glycolysis may be compensating for any drop in ATP levels due to loss of complex I activity. These data are now shown in Supplemental Figure S2H and described in the Discussion on p.12 of the revised manuscript.

The sample size for the ATP assay was 8 controls and 8 ND-75 KD (20 heads per replicate), so it is unlikely that increasing the sample size would change the result. However, in ND-75 KD flies ND-75 levels are not affected in glia and other non-neuronal head cells, which might potentially dilute any changes in ATP. This is why we also analysed ATP levels using the AT-NL fluorescent ratiometric probe in individual ND-75 KD neurons. The AT-NL data also show no change in ATP levels in ND-75 neurons (Supplemental Figure S2A-F), which is consistent with the data from whole heads. We have now included the sample sizes in all figure legends.

Similarly, in Figures S2G-I, the p-AMPK levels in the adult heads signal to a possible increase. The blot should definitely include total AMPK levels and quantification of p-AMPK/AMPK ratio.

We have been unable to obtain convincing immunoblots using a Drosophila total AMPK antibody and so have removed the P-AMPK data from the manuscript.

4) The transcriptomic analysis of head tissue from ND-75KD flies revealed a battery of changed transcripts. Considering the mitochondrial defect and increased ER stress in this model, it would be very useful to further investigate the data in light of classical mitochondrial stress responses, i.e.,

mitochondrial unfolded protein response, antioxidant response, components of mitochondrial integrated stress response, autophagy/mitophagy, etc. Additional bioinformatic analyses concentrating on mitochondrial stress responses would be very beneficial.

We have now performed additional bioinformatic analyses of the RNA-seq data and find little evidence for activation of the mitochondrial UPR, but very good evidence for increased expression of ATF4/ER UPR targets in ND-75 KD fly head tissue. We also find the expression of autophagy and antioxidant genes are mis-regulated ND-75 KD head tissue. These data are described in the revised manuscript on p.8/9 and in additional Supplemental figure panels S3E, F and are documented in the supplemental tables S1-S5 (which now include full details of the GO analyses).

5) Complex I is one of the major reactive oxygen species (ROS) producers in mitochondria (ROS is being used as an umbrella term for superoxide and hydrogen peroxide in this context). NDI1 has also been shown to increase reverse electron transport-mediated ROS (RET-ROS) levels and regulate stress adaptation, lifespan and even mitochondrial function in Drosophila.

As the ND-75KD flies have huge decrease in complex I activity and increased stress responses, measuring ROS levels and some players of antioxidant defenses in the brain before and after NDI1 introduction could add great value to the study.

We have now analysed ROS levels in ND-75 KD neurons before and after NDI1 expression. ND-75 KD causes decreased ROS, while NDI1 expression alone or together with ND-75 KD causes increased ROS. We have also now included data showing that genetic modulation of antioxidant enzymes does not modify the ND-75 KD climbing phenotype. These data indicate that altered ROS does not contribute to neurological dysfunction in ND-75 KD flies. These data are described in the Results on p.11 and in Supplemental Figure S4 of the revised manuscript.

6) The animal models of mitochondrial dysfunction generally present with increased lactate levels. In this case, lactate elevation was not detected in the metabolomics experiment. This point can be discussed.

Although lactate missed the statistical cut-off (FDR-adjusted p value of <0.05) in the analysis of the metabolomic data overall, a student's t test of the lactate levels in control and ND-75 KD fly head tissue showed that lactate levels are significantly increased by ND-75 KD. These data are now described in the Discussion on p.12 and shown in Figure S2H in the revised manuscript.

Stylistic comments:

1) In general, the experimental results of larval and adult knockdowns are hard to distinguish just looking at the figures. It would be useful to have some indication for this separation.

All the data in Figure 2, S2 and S4 are all from larval neurons so for clarity we have now stated this in the title of these figure legends. In Figures 3 and 6, which show both larval and adult data, we have labelled these in the appropriate panels.

2) The sample sizes must be added to the figure legends, where applicable.

We have added sample sizes to all the figure legends.

3) Line numbers should be added to the manuscript as required by the journal.

We have added line numbers.

4) The references should be styled according to the journal submission guidelines.

We have formatted the references according to the PLOS submission guidelines.

5) "… a readout of AMP/ATP ratio (Supplemental Fig. S2G-F)." should be Supplemental Fig. S2G-I.

Thanks for spotting this but these data have now been removed from the revised manuscript.

6) In the Supplementary Table 1, some gene names have been changed into dates in Excel. These should be corrected.

Thanks for spotting this, we have corrected these gene names in Supplemental Table 1.
